# Phyto-Sesquiterpene Lactones Prevent the Development of Multidrug Resistance in TNBC via ABC Transporters Inhibition and STAT3/MYC Signaling

**DOI:** 10.3390/cancers17081321

**Published:** 2025-04-14

**Authors:** Ying-Tzu Chang, I-Ting Wu, Chien-Hsing Lee, Chin-Chuan Hung

**Affiliations:** 1 Department of Pharmacy, College of Pharmacy, China Medical University, Taichung 406040, Taiwan; changyt@365.cmu.edu.tw (Y.-T.C.); u105015202@cmu.edu.tw (I.-T.W.); 2 School of Post-Baccalaureate Medicine, College of Medicine, Kaohsiung Medical University, Kaohsiung 80708, Taiwan; chlee0818@kmu.edu.tw; 3 Department of Pharmacology, School of Medicine, College of Medicine, Kaohsiung Medical University, Kaohsiung 80708, Taiwan; 4 Department of Medical Research, Kaohsiung Medical University Hospital, Kaohsiung 80708, Taiwan; 5 Department of Pharmacy, China Medical University Hospital, Taichung 404327, Taiwan; 6 Department of Healthcare Administration, Asia University, Taichung 41354, Taiwan

**Keywords:** triple-negative breast cancer, multidrug resistance, ABCB1, ABCG2, MYC, STAT3

## Abstract

Background: Multidrug resistance (MDR) in triple-negative breast cancer (TNBC) leads to treatment failure and tumor recurrence. Dysregulation of the MYC oncogene is associated with the pathogenesis of TNBC and the development of chemoresistance via overexpression of ATP-binding cassette (ABC) transporters. Therefore, in the present study, we aimed to identify molecules from a natural product origin that prevent the development of MDR in TNBC by targeting the MYC signaling. Methods: The cell viability of TNBC was evaluated using sulforhodamine assay. Protein levels were detected by western blots or enzyme-linked immunosorbent assays. Intracellular calcein and hoechst33342 accumulation assay aimed to evaluate the inhibitory ability of phytocompounds on drug-efflux functions of *ABCB1* and *ABCG2* transporters. The Cancer Genome Atlas (TCGA) database was used to explore clinical genomic data. Furthermore, the zebrafish xenotransplantation model bearing Dil-labeled TNBC cells was applied to testify the in vivo effects of phyto-sesquiterpene lactones. Results: The results of the present study demonstrated that the phyto-sesquiterpene lactones exhibited an MDR prevention effect by repressing efflux activities of *ABCB1* and *ABCG2* transporters. Mechanistic studies showed that phyto-sesquiterpene lactones inducted TNBC cell apoptosis and cell cycle G2/M arrested by blocking the STAT3/MYC pathway. Clinical genomic data demonstrated that the percentages of MYC amplification and mRNA were upregulated approximately two-fold higher in the TNBC patients than the non-TNBC breast cancer patients. The survival of patients with an alteration in MYC was significantly lower in TNBC as compared to other subtypes. Moreover, the results of the zebrafish xenograft model confirmed that phyto-sesquiterpene lactones exerted stronger inhibitory effects on TNBC tumor growth in vivo. Conclusions: In conclusion, these three phyto-sesquiterpene lactones were promising candidates for TNBC treatment and shed light on the prevention of developing MDR TNBC.

## Simple Summary

Triple-negative breast cancer is an aggressive form of breast cancer that lacks effective targeted therapies, making chemotherapy the primary treatment option. However, many patients develop resistance to chemotherapy over time, limiting its effectiveness. MYC, a key oncogene, and STAT3 signaling have been linked to Triple-negative breast cancer progression and treatment resistance. Additionally, multidrug resistance caused by ATP-binding cassette transporters reduces the effectiveness of chemotherapy drugs. Natural compounds, such as sesquiterpene lactones, have shown potential in targeting cancer cells while minimizing toxicity. Helenalin and its derivatives, bis(helenalinyl)malonate and bis(helenalinyl)glutarate, have demonstrated anticancer properties in other cancers, but their effects on triple-negative breast cancer and multidrug resistance remain unclear. This study aims to evaluate the impact of helenalin, bis(helenalinyl)malonate, and bis(helenalinyl)glutarate on triple-negative breast cancer growth and drug resistance, providing insights into their potential as novel therapeutic options.

## 1. Introduction

Nearly 15% of invasive breast cancers are devoid of estrogen receptor (ER), progesterone receptor (PR), and human epidermal growth factor receptor type 2 (HER2) expression and, thus, defined as triple-negative breast cancer (TNBC) [1]. It is an aggressive subtype with a poorer prognosis compared to other types of breast cancer [2]. Since targeted therapies are weakly effective for TNBC, the main therapeutic method is systemic chemotherapy, such as anthracyclines and/or taxanes. However, most patients often become chemoresistant within 3–4 years. Novel therapeutics with poly (ADP-ribose) polymerase (PARP) inhibitors are indicated for patients who carry BRCA mutation and are a promising therapeutic approaches when combined with chemotherapeutic agents in TNBC [3]. Despite the great potential of PARP inhibitors, several mechanisms have been identified that contribute to resistance toward the PARP inhibitors or other chemotherapeutic agents, such as altered protein expression, target mutations, increased DNA repair, changes in drug metabolism, or the presence of multidrug-resistance (MDR) transporters [4,5]. Novel therapeutic options for TNBC patients are urgently needed.

Dysregulation of the MYC oncogene is associated with the pathogenesis of many human cancers. Elevated MYC expression is often detected in TNBC and related to poor outcomes [6,7,8,9]. MYC, a proto-oncogene, plays a pivotal role in the cell growth and survival processes of cancers, such as tumor metastasis, cell metabolism, and DNA repair [10]. This suggests the MYC-driven oncogenic transformation of cancers, resulting in dependence on MYC expression. In addition, studies have revealed that signal transducer and activator of transcription 3 (STAT3) promotes the proliferation of TNBC and apoptosis arrestment by increasing the level of its downstream target, MYC [11]. Mounting evidence suggested that STAT3-mediated MYC overexpression is important in various malignancies [12,13]. Multidrug resistance is another critical obstacle for TNBC clinical therapeutic. More and more evidence suggests that chemotherapy drug resistance is driven through the overactivity of ATP-binding cassette (ABC) transporters [14]. Among these ABC transporters, P-glycoprotein (P-gp, MDR1, *ABCB1*) and breast cancer resistance protein (BCRP, *ABCG2*) are the main drug-efflux proteins in clinically important cases of MDR [15]. It was well established that tumor cells with high expression of ABC efflux transporters decrease the amount of intracellular chemotherapy drugs, including TNBC standard treatments, paclitaxel, docetaxel, and doxorubicin [16]. The preceding literature demonstrated that the upregulation of *ABCB1*/MDR1 was induced after docetaxel or anthracycline/taxane-based chemotherapy treatment in TNBC cells [17,18]. The *ABCG2* expression was positively associated with proliferative index, Ki67, in TNBC patients [19]. Evidence suggests that the development of novel therapies targeting MYC dysfunction may provide a solution for TNBC.

Considering all possible natural compounds that are used in medicine, sesquiterpene lactones could be proposed as promising molecules for cancer therapy. Sesquiterpene lactones are secondary plant metabolites with potent bioactivities, including anti-inflammation, antioxidant, and cytotoxicity [20,21]. In TNBC studies, sesquiterpene lactones specifically inhibit TNBC cell lines without inhibiting normal breast cells and exert anticancer activity via suppressing STAT3 or inducing cell cycle arrest, apoptosis, and autophagy [22,23,24,25]. Helenalin, a sesquiterpene lactone, obtained from *Helenium microcephalum* [26], exhibits cytotoxic ability on a variety of cancers by inhibiting DNA synthesis and cellular enzyme activity, as well as its derivatives, bis(helenalinyl)malonate (BHM) and bis(helenalinyl)glutarate (BHG) [27,28,29]. The synthesis of bis(helenalinyl) esters aims to clarify the bioactivity of β-unsubstituted cyclopentenone and α-methylene γ-lactone. Two bifunctional alkylated sesquiterpene lactones are combined by diester bonds to improve antitumor activity while reducing toxicity [28]. Whether helenalin, BHM, and BHG influence the TNBC proliferation and MDR development has not been investigated. Hence, the present study aimed to investigate the effects of helenalin, BHM, and BHG on the TNBC and MDR phenomenon. Further, the underlying molecular mechanisms were comprehensively explored in in vitro and in vivo studies.

## 2. Materials and Methods

### 2.1. Chemicals and Reagents

**Figure 1:**
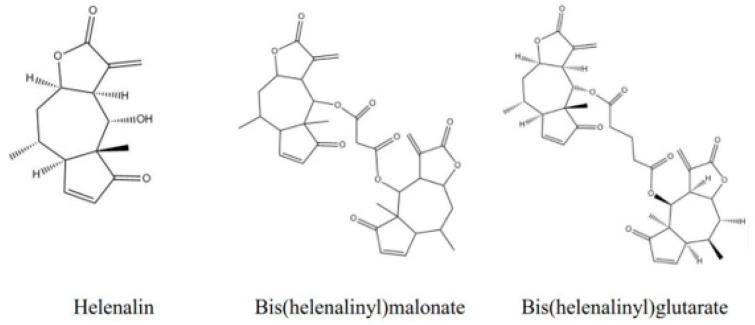
Structure of helenalin, BHM, and BHG.

The following chemicals were obtained from Sigma Chemical Co. (St. Louis, MO, USA): paclitaxel, docetaxel, doxorubicin, R-(+)-verapamil, fumitremorgin C (FTC), menadione, JQ1, calcein-AM, hoechst33342, phosphate-buffered saline (PBS), sulforhodamine B (SRB), trichloroacetic acid (TCA), and Tris Base. The culture medium, DMEM and RPMI, were purchased from Thermo Fisher Scientific Inc., Waltham, MA, USA. Helenalin, bis(helenalinyl)malonate (BHM), and bis(helenalinyl)glutarate (BHG) were obtained from BenchChem (Pasadena, CA, USA) with 98%, 95%, and 95% purity, respectively. The structures are shown in Figure 1.

### 2.2. Cell Culture

The human MYC expressing TNBC cell line MDA-MB-231 [10], MDA-MB-361 (ER+), MCF7 (ER+), MDA-MB-453 (HER2+) breast cancer cell lines, and other cancer cells, HepG2 and NCI-H460, were obtained from the Bioresource Collection and Research Center (BCRC) in Taiwan. MDR TNBC cell line, MDA/doc, was selected from MDA-MB-231 with gradually increased concentrations of docetaxel. The human P-gp *(ABCB1*/HEK293) and BCRP (*ABCG2*/HEK293) stable expression cell lines were confirmed by real-time RT-PCR and western blotting. Cells were cultured in Dulbecco’s Modified Eagle Medium (DMEM) or Roswell Park Memorial Institute (RPMI) 1640 medium supplemented with 10% fetal bovine serum, under standard conditions of 37 °C, 95% relative humidity, and 5% CO_2_ [30].

### 2.3. Cell Viability Assay

Cytotoxicity after each compound treatment was detected through the SRB assay, as previously mentioned [30]. Cells were seeded in plates with 72-h treatments, then living cells were fixed and stained with 50% TCA and 0.04% SRB, respectively. Ultimately, 10 mM of tris-base solubilized the bound stain, which was then measured using a multimode microplate reader at 515 nm.

### 2.4. Cell-Cycle Analysis

MDA-MB-231 cells were starved in a serum-free medium overnight and then cultured with the compounds. After 72 h, cells were collected in freezing 70% ethanol. The fixed cells were stained using propidium iodide (PI) overnight, followed by evaluation by a flow cytometry system with excitation 488 nm and emission 575 nm (BD FACSCanto II System, BD Biosciences, Franklin Lakes, NJ, USA).

### 2.5. Apoptosis Assay

Apoptosis was analyzed in line with the instruction of the reagent (BD Pharmingen™, San Diego, CA, USA, Catalog No. 556547). After 72-h treatments, MDA-MB-231 cells were collected and dyed with Annexin V and PI (protected from light). All the cells were determined by a flow cytometry system with excitation 488 nm and emission 530/575 nm (BD FACSCanto II System).

### 2.6. Reactive Oxygen Species (ROS) Level Detection

The ROS modification after compound treatment was evaluated according to the instructions of the ROS detection kit (AAT Bioquest^®^, Inc., Pleasanton, CA, USA). MDA-MB-231 cells were treated with compounds (menadione was used as a positive control) for 2 h to induce ROS. The fluorescence was measured by a multimode microplate reader (Ex: 490 nm, Em: 525 nm).

### 2.7. Enzyme-Linked Immunosorbent Assay (ELISA)

After 72 h compound treatment, MDA-MB-231 cells were collected and lysed by RIPA Lysis and Extraction Buffer (Thermo Scientific™). Standard ELISA assays were performed to determine the level of human cyclin dependent kinase 1 (CDK1), MYC, *p21* (Elabscience^®^, Houston, TX, USA, Catalog No: E-EL-H2321, E-EL-H0756, and E-EL-H1740), cell division cycle protein 25 (CDC25, Mybiosource, San Diego, CA, USA, Catalog No: MBS452073), and STAT3-pY705 (Abcam^®^, Boston, MA, USA, Catalog No: ab126458).

### 2.8. siRNA Gene Silencing

MDA-MB-231 cells were transfected with either MYC-specific siRNAs (Ambion, Foster City, CA, USA, 5′-AGACCUUCAUCAAAAA-3′, Catalog No: s9129; 5′-GAGCUAAAACGGAGCUUUU-3′, Catalog No: s9130) or equivalent scrambled siRNA control through transfection reagent (Thermo Scientific™). After 48 h of gene silencing, the MYC expression was examined by western blotting analysis. The transfected cells were used to verify the role of MYC gene via SRB assay.

### 2.9. Western Blot Assays

MYC expression levels were quantified by western blotting analysis. After a 72-h treatment, the protein expression levels were detected using the iBind™ Western System (Thermo Scientific™). Cell lysates were subjected to the Bolt™ 10% Bis-Tris Plus Gels, and then the proteins were separated to transfer onto polyvinylidene difluoride (PVDF) membrane (Thermo Scientific™). The membranes were incubated with antibodies to MYC (GTX628259) and β-actin for 4 h. Lastly, protein levels were imaged by the Luminescent Image Analyzer LAS-4000 (GE Healthcare, Chicago, IL, USA).

### 2.10. P-gp and BCRP Fuctional Assay

The influence of helenaline, BHM, and BHG on P-gp and BCRP efflux activity were assessed by detecting the fluorescence of calcein and hoechst33342 in cells as previously mentioned [30]. In simple terms, *ABCB1*/HEK293 cells, *ABCG2*/HEK293, and HEK293 cells were treated with each compound and then incubated with the calcein-AM (substrate of P-gp) or hoechst33342 (substrate of BCRP) for 30 min. The intracellular fluorescence was detected by a multimode microplate reader with excitation 485 nm and emission 528 nm for calcine, excitation 340 nm and emission 460 nm for hoechst33342.

### 2.11. Compound–Drug Combination Assay

The synergistic cytotoxic effects of helenalin, BHM, and BHG with chemotherapeutic drugs were investigated in *ABCB1*/HEK293 and *ABCG2*/HEK293 cells, as well as in the MDR MDA/Doc cells. Cells were incubated with chemotherapeutics only or co-treated with compounds for 72 h; the IC_50_ values were evaluated by SRB assay.

### 2.12. Synergism Analysis

To assess the synergistic effects of helenalin, BHM, and BHG in combination with various chemotherapeutic agents, we utilized the computational platform SynergyFinder Plus in conjunction with the Highest Single Agent (HSA) reference model. Synergy scores were interpreted according to standard criteria: scores below −10 indicate antagonism, scores between −10 and 10 suggest additive effects, and scores greater than 10 reflect synergistic interactions.

### 2.13. Genomic Data Analysis from Breast Cancer Dataset

Clinical datasets were used to analyze the alterations of MYC and MYC-related cell survival pathways in TNBC patients. Clinical patient profiling was retrieved from Breast Invasive Carcinoma (TCGA provisional, *n* = 1105) and Breast Cancer (METABRIC, *n* = 2509) datasets with cBioPortal (http://www.cbioportal.org/).

### 2.14. Zebrafish Xenograft Assay

Healthy zebrafish (Danio rerio) were purchased from the Taiwan Zebrafish Core Facility at Academia Sinica (TZCAS, Taipei, Taiwan) to appraise the in vivo efficacy and safety of helenalin, BHM, and BHG in the MDA-MB-231 zebrafish xenograft model. The maintenance and animal experiments of zebrafish were handled in conformity with the guidelines by the ethics committee of the Institutional Animal Care and Use Committee (IACUC) and the principles of 3Rs (Reduction, Replacement and Refinement) of Kaohsiung Medical University (Kaohsiung Medical University, Kaohsiung, Taiwan). Zebrafish were maintained in aquariums at 28.5 °C and maintained with a 10 h dark and 14 h light period as before [31]. Zebrafish embryos were anesthetized 48 h post-fertilization (hpf) with 0.01% tricaine, and MDA-MB-231 cells, which were marked with Dil stain (1,1′-Dioctadecyl-3,3,3′,3′-Tetramethylindocarbocyanine Perchlorate, Molecular Probes, Carlsbad, CA, USA), were implanted into zebrafish embryos, and the growth of tumors was tracked using fluorescence microscopy. Each of embryos was treated with helenalin, BHM, BHG, and paclitaxel (as a positive control) at the indicated concentrations for 24 and 48 h post-injection (hpi). Subsequently, the tumor size in each zebrafish was evaluated by inverted microscope (Nikon Eclipse TE2000-U, Tokyo, Japan).

### 2.15. Statistical and Data Analysis

The half-maximal inhibitory concentration, IC_50_ value, was used to assess the inhibitory effect of test compounds and calculated as the following formula:

$$E=E_{0}\left(\frac{IC50^{s}}{IC50^{s}+I^{s}}\right)$$

E and E_0_ represented the inhibitory ability with and without compound treatment. The concentration of inhibitors was denoted as I, and the half maximal inhibitory concentration of test compounds was identified by IC_50_. S was the slope factor. Statistical differences in all data were measured using post hoc analysis (Tukey’s test) or Student’s *t*-test with ANOVA. If the *p*-value is less than 0.05, it is defined as statistically significant.

## 3. Results

### 3.1. The MDR Prevention Effect of Phyto-Sesquiterpene Lactones Through Inhibiting ABC Transporters

**Figure 2:**
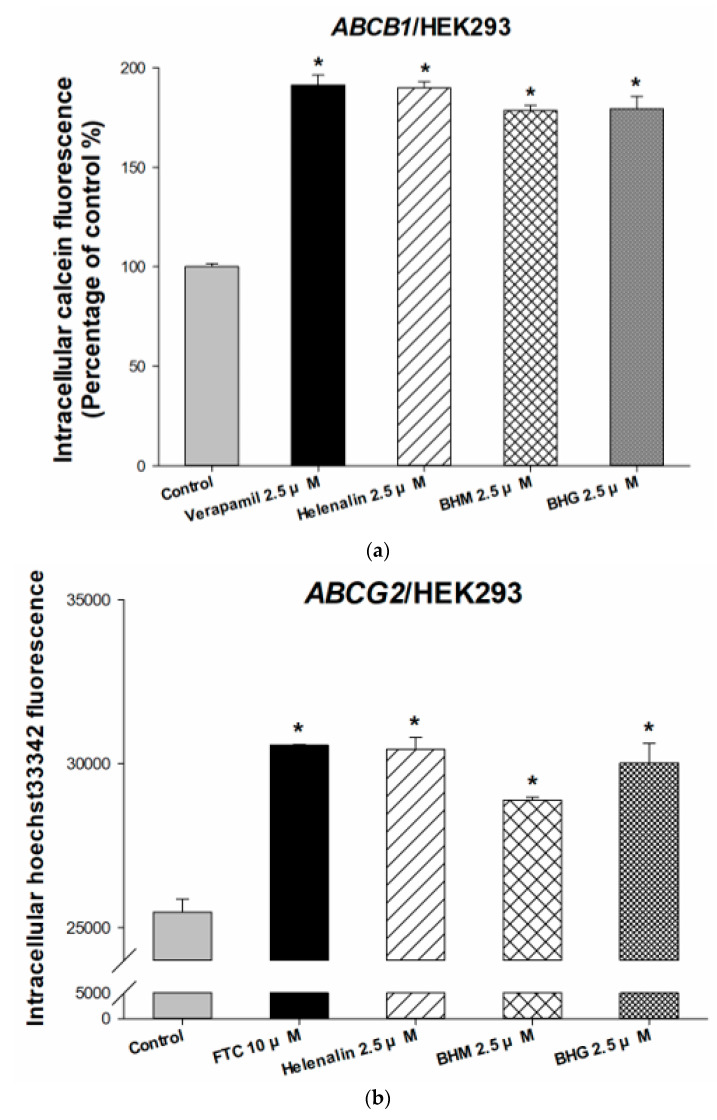

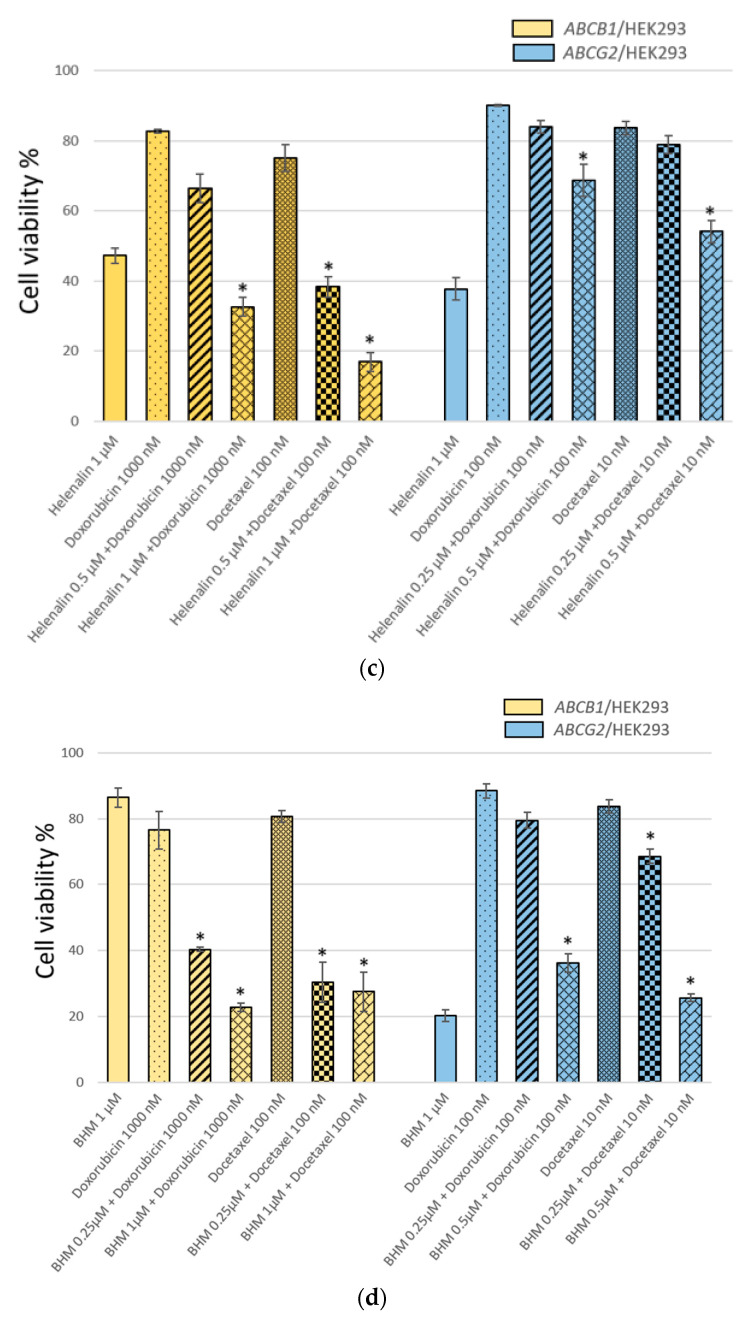

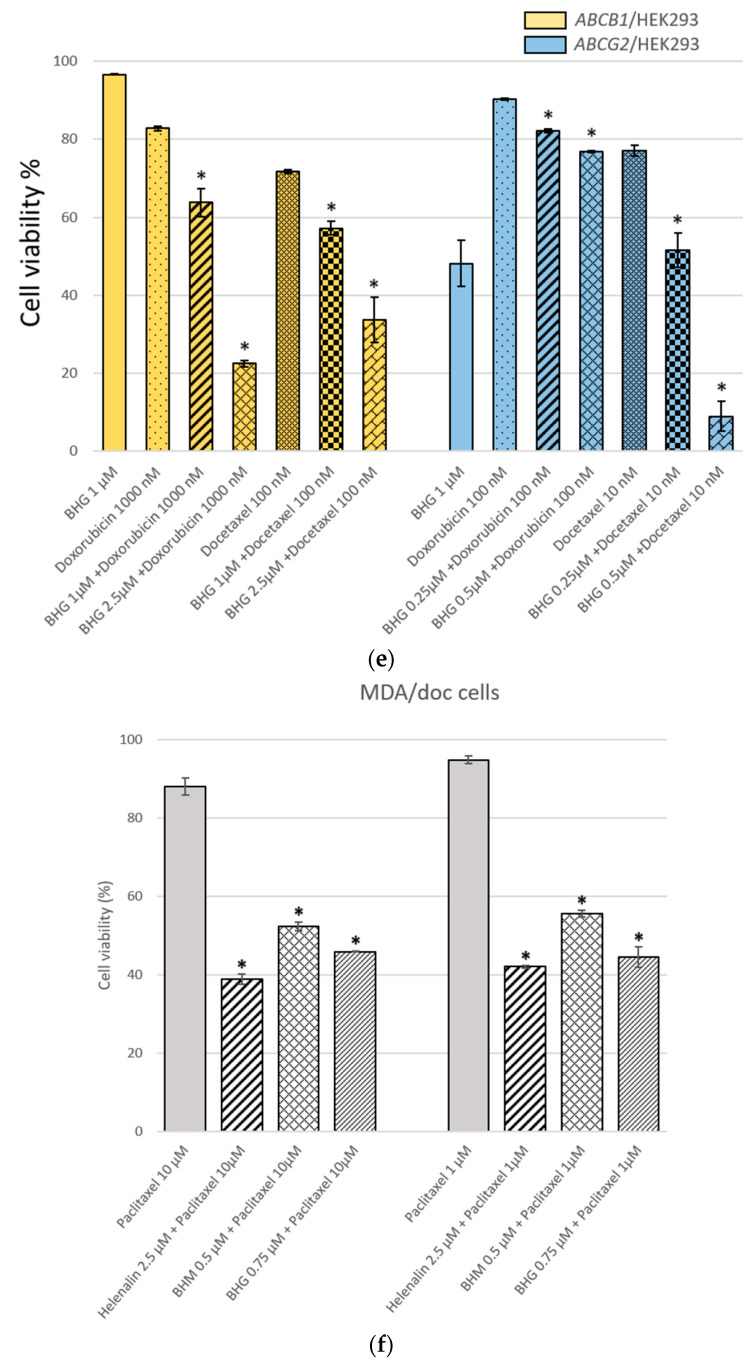

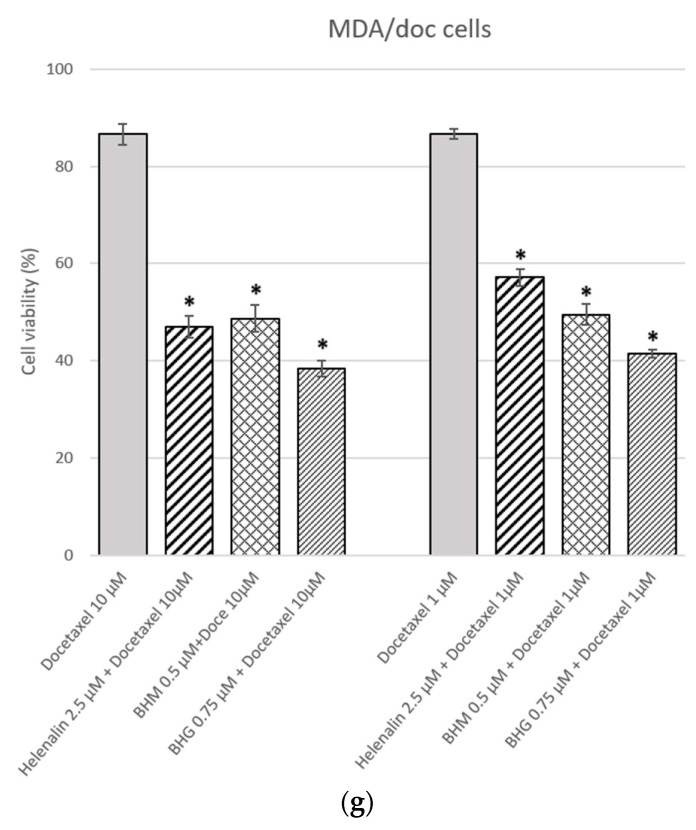

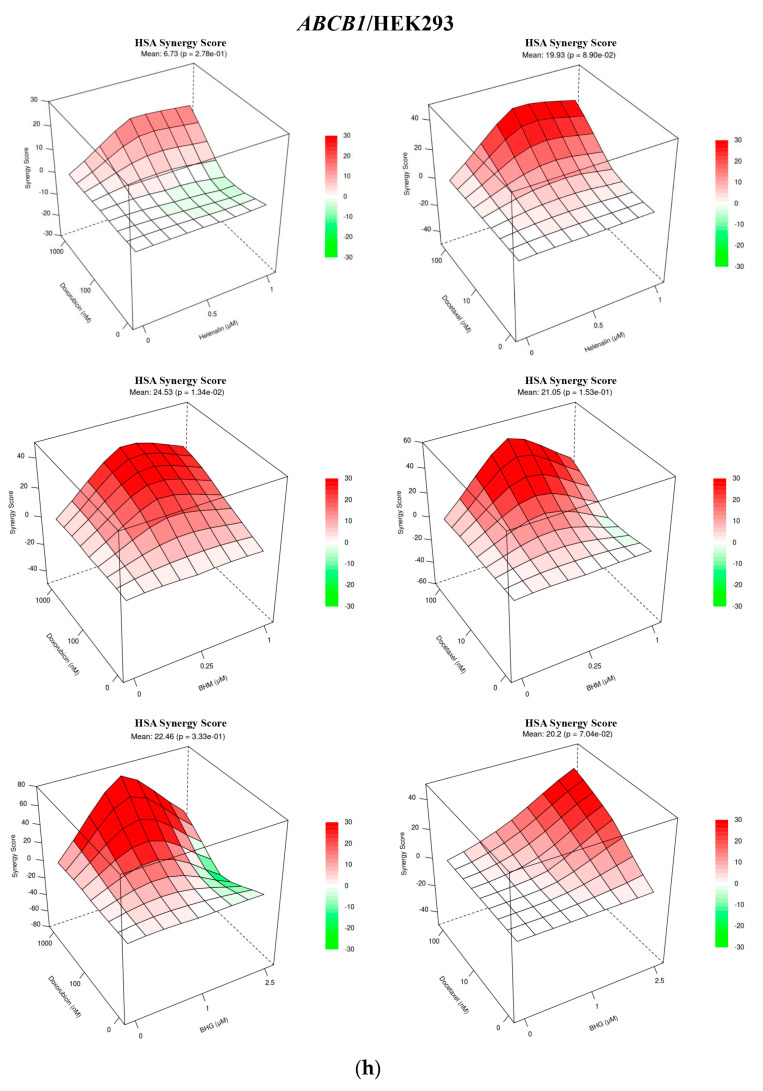

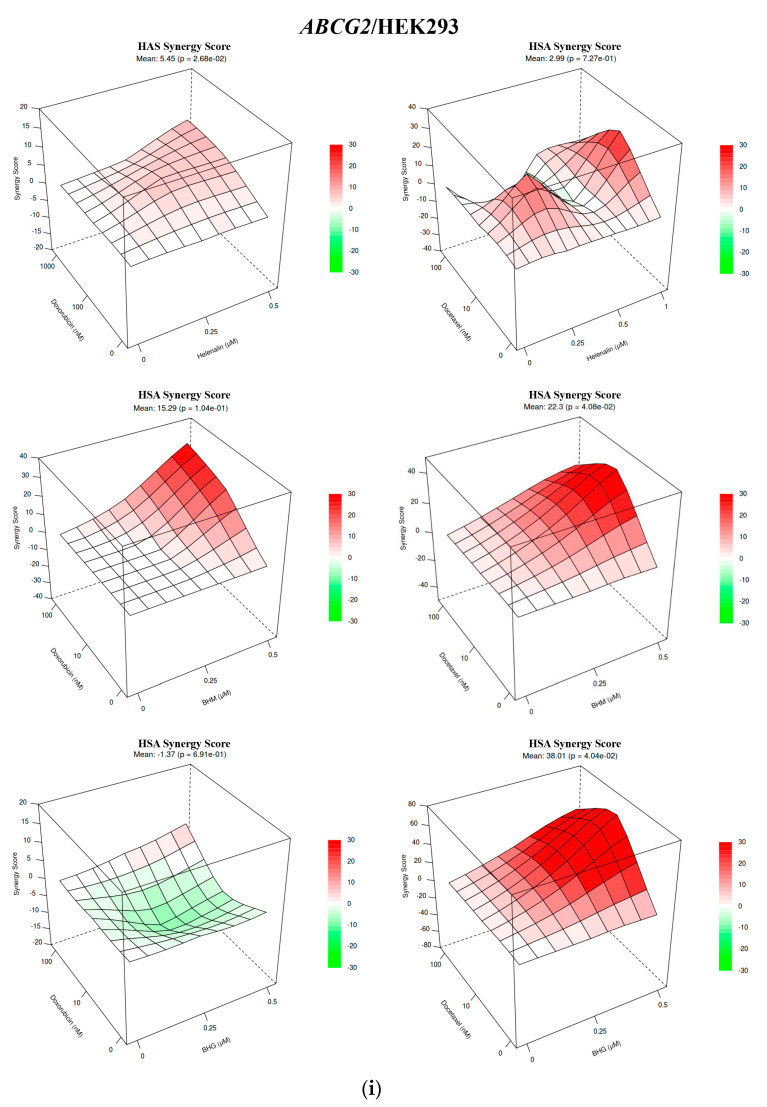
Inhibition abilities and chemosensitizing effects of helenalin, BHM, and BHG on ABC transporters in *ABCB1* or *ABCG2* overexpressing cells. (**a**,**b**) The intracellular calcein and hoechst33342 fluorescence were increased under helenalin, BHM, or BHG 30 min treatments in *ABCB1*/HEK293 cells and *ABCG2*/HEK293 cells. (**c**–**e**) The synergistic cytotoxicity of helenalin, BHM, or BHG co-treated with docetaxel and doxorubicin indicated that helenalin, BHM, or BHG increased sensitivity to the chemotherapeutics docetaxel and doxorubicin in *ABCB1*/HEK293 cells and *ABCG2*/HEK293 cells. (**f**–**g**) The MDR preventive effects of helenalin, BHM, and BHG in MDA/doc cells. Synergy assessment of helenalin, BHM, and BHG in combination with doxorubicin or docetaxel in *ABCB1*/HEK293 (**h**) and *ABCG2*/HEK293 (**i**) cell lines. Synergy scores were interpreted as follows: <−10, antagonism; −10 to 10, additive effect; >10, synergism. Statistical differences were evaluated by the ANOVA followed by post hoc analysis (Tukey’s test). * denotes *p* < 0.05 as compared to doxorubicin or docetaxel alone treatments. Each data is expressed as the mean ± standard error of at least two experiments, each performed in duplicate.

Multidrug resistance is a critical challenge for TNBC patients after receiving chemotherapy, which is caused primarily by the overexpression of ABC efflux transporters. Previous studies have shown that *ABCB1*/MDR1 and *ABCG2*/BCRP are positively associated with chemoresistance and cell proliferation in TNBC. Therefore, the modulation effects of test compounds on *ABCB1* and *ABCG2* transporters were detected by intracellular calcein and hoechst33342 accumulation assays. Calcein and hoechst33342 are the fluorescence substrates of the *ABCB1* transporter and *ABCG2* transporter, respectively. With helenalin, BHM, or BHG treatment, the intracellular fluorescence was increased, which indicated that the test compounds significantly inhibited the efflux function of the *ABCB1* and *ABCG2* transporters (Figure 2a,b). To investigate the MDR-reversal effect, we compared the cytotoxicity of conventional chemotherapeutic only with those treated with combination therapy. The compound-drug combinations showed synergistic effects on attenuated cell viabilities in the *ABCB1* and *ABCG2* overexpressing cells, and the effects were dose-dependent (Figure 2c–e). We further revealed the MDR preventive ability of helenalin, BHM, and BHG on TNBC MDR cells. The resistance fold was 1443.62, which was calculated by the dividing IC_50_ of MDR cells (MDA/doc) by the IC_50_ of parental cells (MDA-MB-231). Figure 2f,g demonstrated that helenalin, BHM, and BHG significantly restored the sensitivity of chemotherapeutic drugs in MDR TNBC cells. The above results indicated that these phyto-sesquiterpene lactones could prevent the development of MDR in TNBC through inhibiting ABC transporters. A synergism analysis was performed to evaluate the combinatorial effects of helenalin, BHM, and BHG with conventional chemotherapeutic agents. In line with the observed cytotoxicity profiles, the calculated synergy scores indicated that co-treatment with doxorubicin or docetaxel resulted in synergistic or additive interactions when combined with helenalin, BHM, or BHG in both *ABCB1*/HEK293 and *ABCG2*/HEK293 cell lines (Figure 2h,i).

### 3.2. MYC Suppressing Effect of Phyto-Sesquiterpene Lactones on TNBC Cells

The different types of cancer cells were treated with compounds at various concentrations to explore the antiproliferative effect of helenalin, BHM, and BHG on human TNBC cells. Compared with other cancers hepatocarcinoma HepG2 cells, and non-small cell lung cancer NCI-H460 cells, even within other subtypes of breast cancer MDA-MB-361, MCF7, and MDA-MB-453, the TNBC cell line MDA-MB-231 cells exhibited significantly higher cytotoxic-sensitivity to helenalin, BHM, and BHG with IC_50_ values 0.63, 0.07, and 0.55 μM, respectively (Table 1). Furthermore, helenalin, BHM, and BHG demonstrated better cytotoxic effects toward MDA/Doc cells than paclitaxel (Table 1).

**Figure 3:**
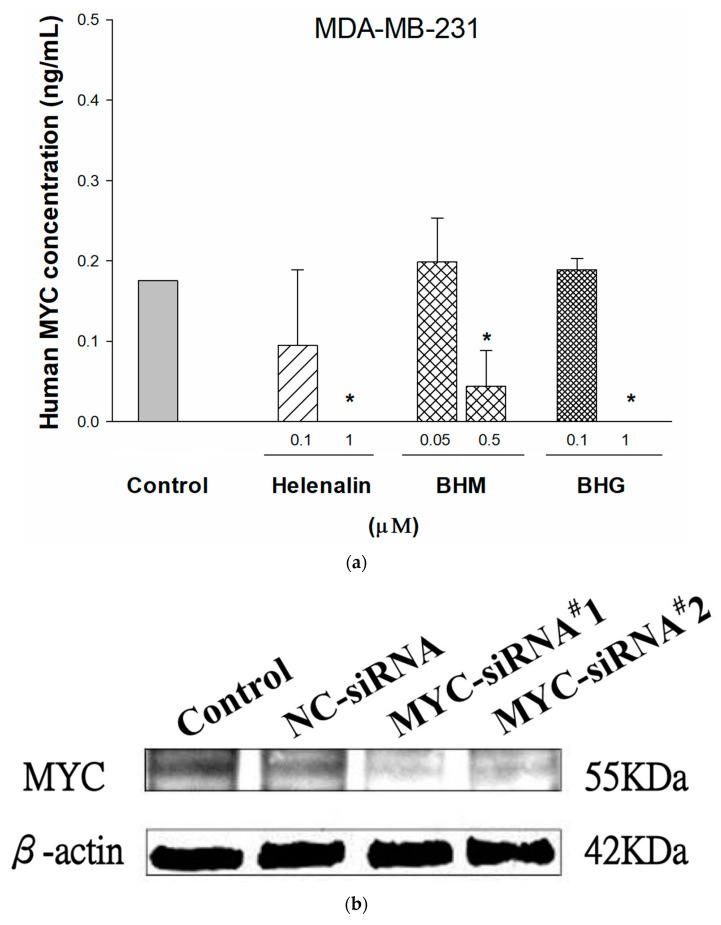

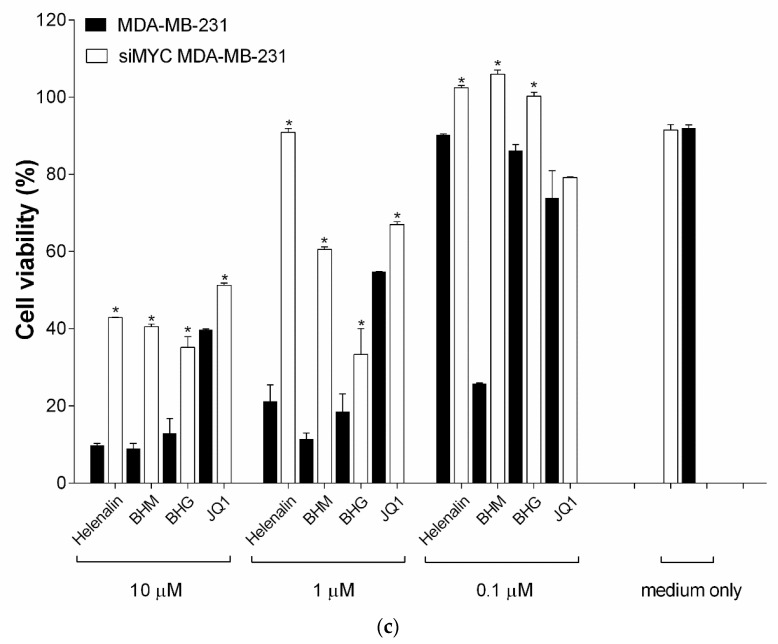
The effects of helenalin, (helenalinyl)malonate (BHM), and bis(helenalinyl)glutarate (BHG) on cell proliferation of MDA-MB-231 through MYC-dependent pathway. (**a**) MYC expression under helenalin, BHM, and BHG 72 h treatments in MDA-MB-231 cells was determined by enzyme-linked immunosorbent assay (ELISA). (**b**) Relative MYC levels in MDA-MB-231 cells transfected with scrambled siRNA (negative control) or MYC siRNA by western blotting. The uncropped blots are shown in the Supplemental Materials. (**c**) The cytotoxic effect of helenalin, BHM, and BHG in MDA-MB-231 cells with or without MYC siRNA transfection. Cytotoxicity was detected after a 72-h treatment. JQ1 was used as a positive control of MYC inhibitor. Statistical differences were evaluated by Student’s *t*-test. * denoted *p* < 0.05 as compared to untreated control in (**b**) and as compared to MDA-MB-231 cell line in (**c**). Data presented as mean ± standard error of at least two experiments, each in duplicate.

Next, we evaluate the repressive ability of helenalin, BHM, and BHG against MYC expression in vitro; TNBC cells were treated with helenalin, BHM, or BHG at concentrations around IC_50_ values for 72 h. Our results demonstrated that helenalin, BHM, and BHG treatments inhibited MYC expression in MDA-MB-231 cells (Figure 3a). Further confirming the hypothesis that helenalin, BHM, and BHG suppress TNBC cell growth via targeting MYC, we silenced MYC expression by siRNA to determine whether the effect of test compounds was changed. The scrambled siRNA (negative control) or MYC siRNA was transfected into MDA-MB-231, and the expression was measured by western blotting (Figure 3b). The cytotoxic effects of helenalin, BHM, and BHG were decreased as well as the commercial MYC inhibitor, JQ1, in cells stably expressing siRNA against MYC (Figure 3c). These data supported our hypothesis that MYC expression may be a major driver of cell proliferation in human TNBC cells, and helenalin, BHM, and BHG suppressed the tumor cells via targeting MYC.

### 3.3. Phyto-Sesquiterpene Lactones Induced Cell-Cycle Arrest or Apoptosis Through MYC Related Pathway in TNBC Cells

**Figure 4:**
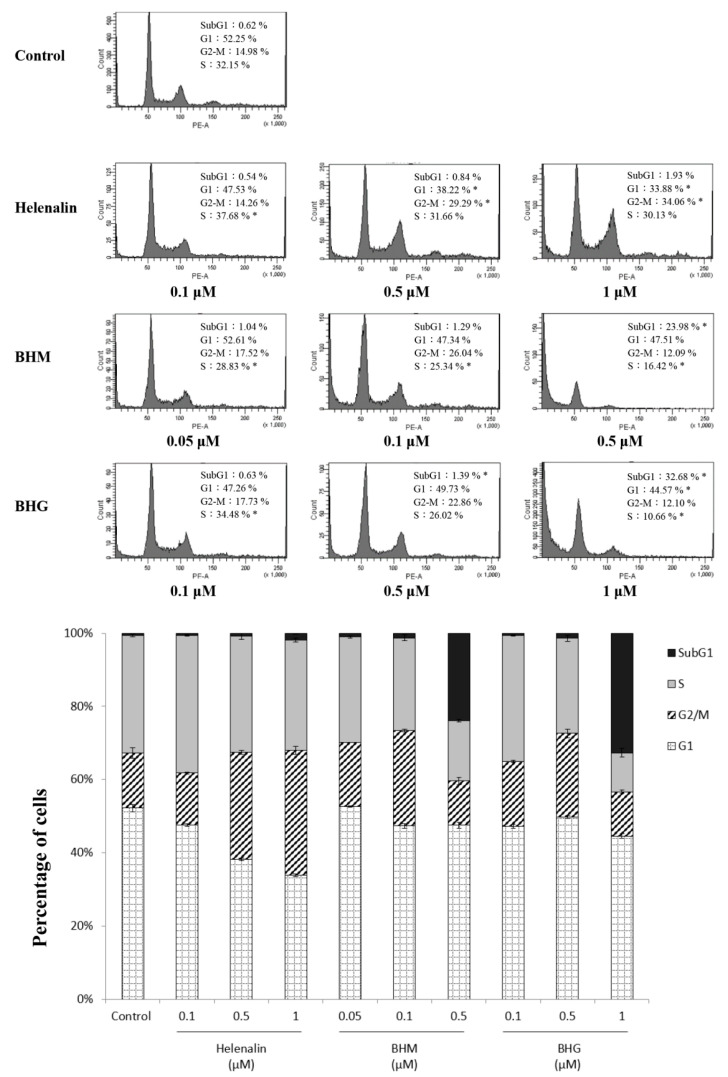
The effects of helenalin, BHM, and BHG on cell cycle in MDA-MB-231 cells. Helenalin, BHM, and BHG significantly induced MDA-MB-231 cell arrest in the G2/M phase and subG1 phase, respectively. * represented *p* < 0.05 as compared to untreated control.

**Figure 5:**
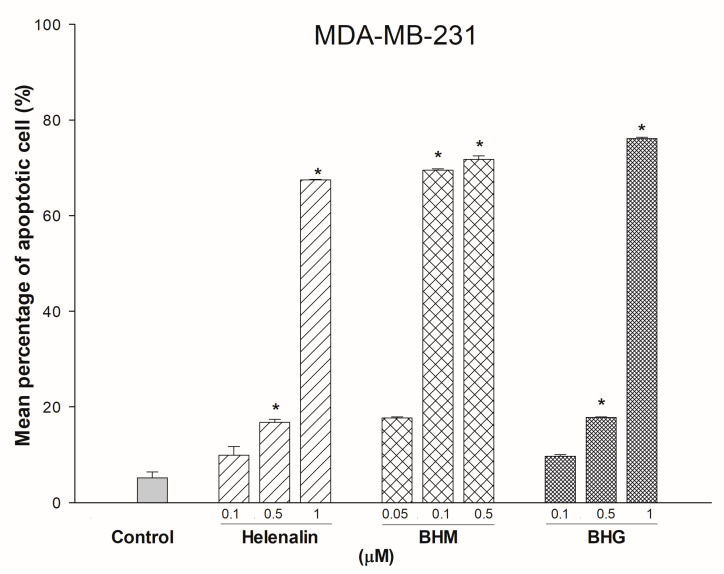
The effects of BHM, and BHG on MDA-MB-231 cells apoptosis. All three test compounds increased apoptotic cells in MDA-MB-231. * represented *p* < 0.05 as compared to untreated control.

We determined the regulation of helenalin, BHM, and BHG on the cell cycle of MDA-MB-231 cells. The results showed that helenalin (a 72-h treatment) increased the G2/M-phase DNA content from 14.98% (control) to 34.06% in MDA-MB-231 cells, whereas BHM and BHG significantly increased the apoptotic sub-G1 DNA content from 0.54% to 23.98% and 32.68%, respectively (Figure 4). We further confirmed the apoptosis induction of the test compounds. Helenalin, BHM, and BHG treatment increased the apoptosis levels of MDA-MB-231 up to 67.5%, 71.75%, and 76.1%, respectively (Figure 5).

**Figure 6:**
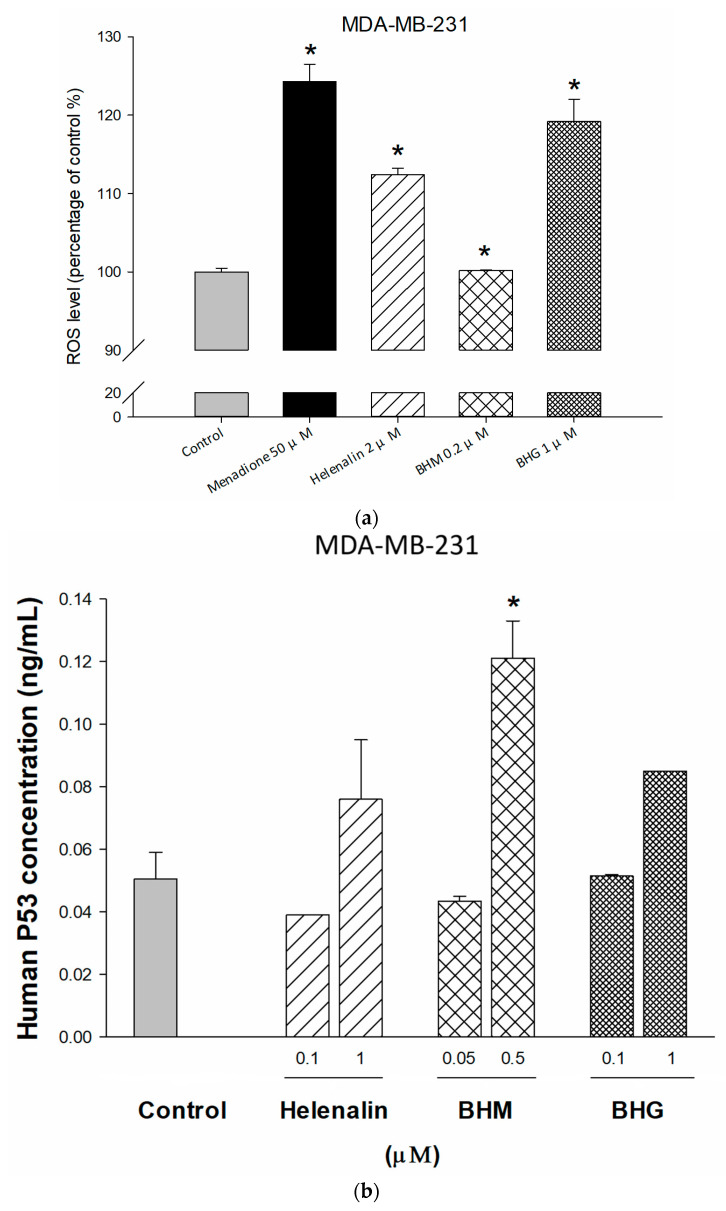

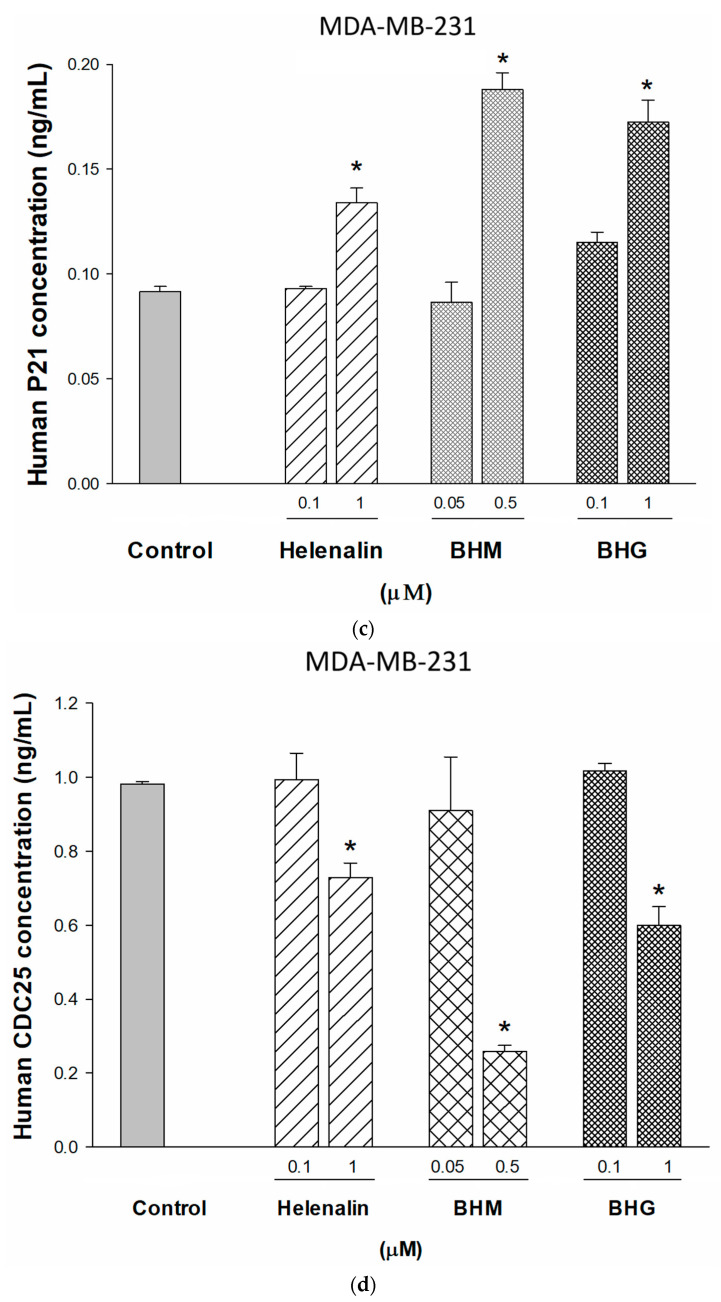

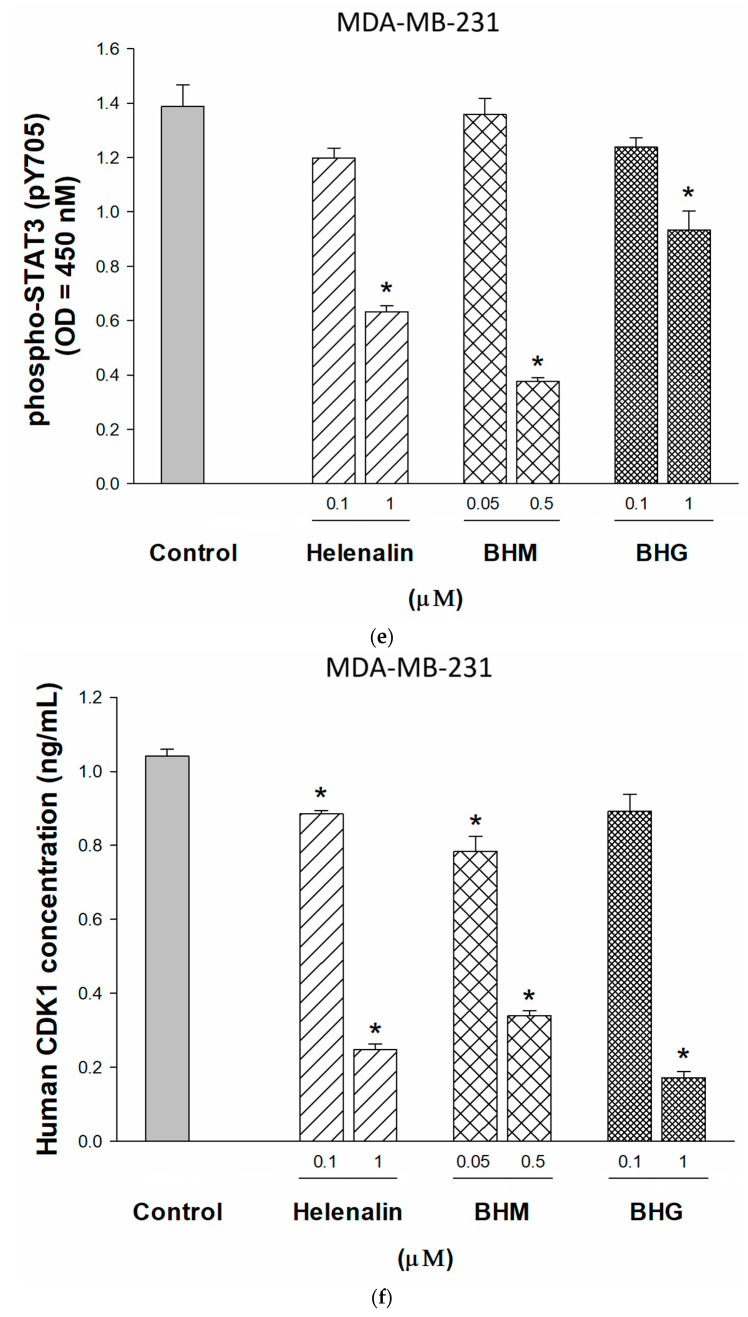
The underlying mechanism of apoptosis and G2/M arrest was determined by ROS assay and ELISA. The apoptotic cell-induced effects of helenalin, BHM, and BHG were detected by increasing ROS levels (**a**) and *p53* expression (**b**). Helenalin, BHM, and BHG suppressed STAT3-MYC-CDC25-CDK1 signaling and increased *p21* to induce G2/M arrest in MDA-MB-231 cells (**c**–**f**). * represented *p* < 0.05 as compared to untreated control. Each data is expressed as the mean ± standard error of at least two experiments, each performed in duplicate.

Since MYC encodes transcription factors with oncogenic roles in many cancers, we further explored the underlying mechanisms of helenalin, BHM, and BHG on MYC signaling. Treatment with helenalin and BHG significantly raised ROS levels (Figure 6a). In addition, *p53* is a tumor suppressor, which mediates cell-cycle arrest or apoptosis through stress stimulation for DNA repair. The *p53* repressed the transcription of MYC by binding to its promoter [32]. Our result showed that elevated levels of *p53* resulted from BHM treatment (Figure 6b). The *p21* protein is a cyclin-dependent kinase inhibitor; the activation of p53 upregulates p21, leading to cell cycle arrest. In a recent study, MYC regulated *p21* transcription and resulted in G2/M cell-cycle arrest [33]. Figure 6c revealed that helenalin, BHM, and BHG induced *p21* expression in MDA-MB-231 cells. Furthermore, STAT3 promoted tumor cell proliferation and suppressed apoptosis and cell cycle progression in TNBC cells by potentiating the expression of MYC and its downstream target genes. MYC also activated the G2/M phase transition protein, cyclin-dependent kinase 1 (CDK1), by upregulating the cell division cycle protein 25 (CDC25) and downregulating *p21*, resulting in G2/M arrest [12,33]. Our data showed that significant inhibition of STAT3, CDC25, and CDK1 in the compound-treated groups (Figure 6d–f).

### 3.4. Bioinformatic Analyze the Role of STAT3-MYC Pathway on TNBC

**Figure 7:**
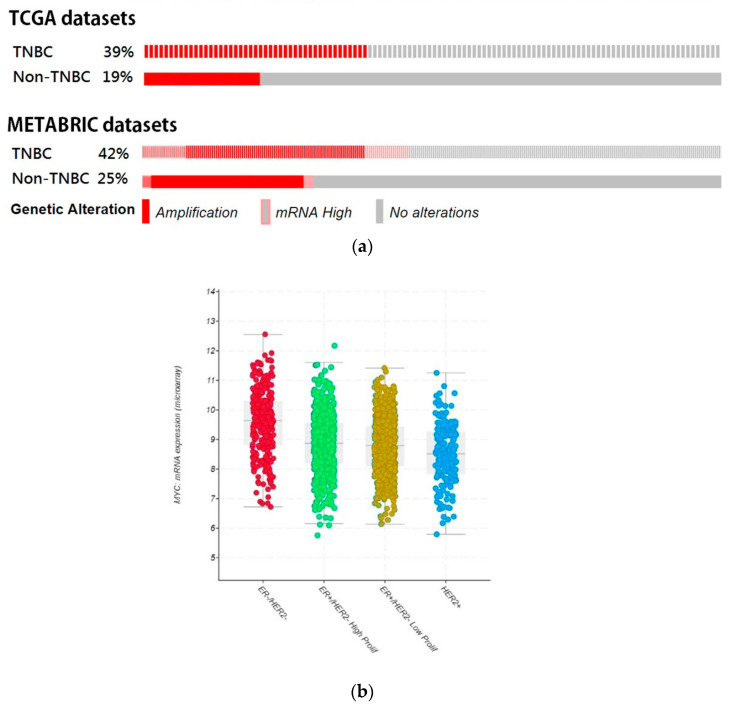

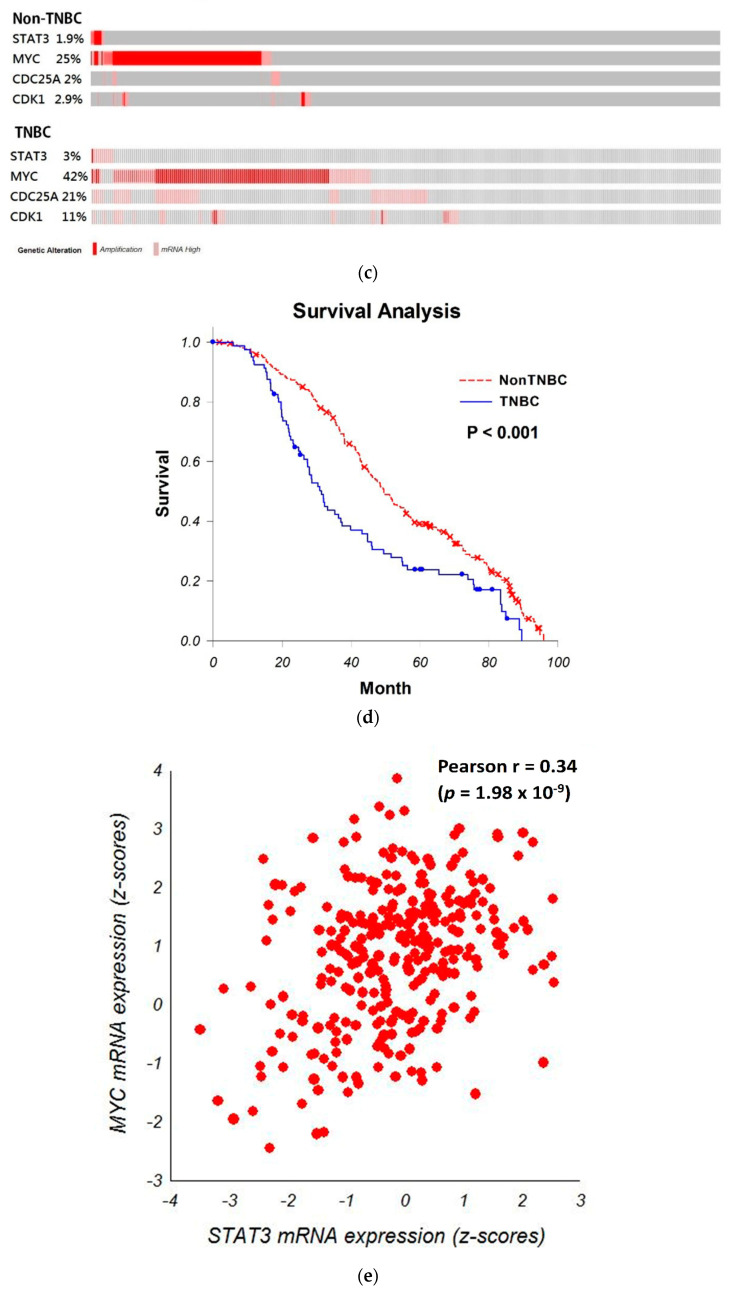
Bioinformatics analysis of the expression of MYC and its regulating pathway in the TNBC patients. (**a**) The oncoprint analysis of MYC amplification or mRNA upregulation in the TNBC cohort and non-TNBC cohort from TCGA and METABRIC breast cancer datasets. (**b**) MYC mRNA expression was detected in breast cancer according to the 3-gene classifier subtype. (**c**) The oncoprint analysis of STAT3, MYC, CDC25, and CDK1 in the TNBC cohort and non-TNBC cohort from METABRIC breast cancer datasets. (**d**) Kaplan–Meier survival analysis of patients with alterations in query genes. (**e**) Pearson’s correlation of STAT3 and MYC expression in the TNBC cohort.

Clinical database analysis was performed to confirm the authenticity and feasibility of our in vitro results of the present study. Figure 7a showed that the percentages of MYC amplification and mRNA upregulation in the TNBC group were approximately two-fold higher than the non-TNBC group (TCGA provisional: 39% in the TNBC group, 19% in the non-TNBC group; METABRIC 42% in the TNBC group, 25% in the non-TNBC group). The gene profiling of patients with breast cancer revealed that MYC mRNA expression was higher in the TNBC (ER-/HER2-) subtype (Figure 7b). Furthermore, the TNBC patients with STAT3, MYC, CDC25, and CDK1 mutational status were approximately two-fold higher than the non-TNBC group (Figure 7c); the survival of patients with alteration in these query genes were significantly lower in the TNBC group as compared to the non-TNBC group (Figure 7d). The correlation of STAT3 and MYC was evaluated by Pearson’s correlation analysis; the coefficient (r) was 0.34 (*p*-value = 1.98 × 10^−9^), which indicated a positive association between STAT3 and MYC mRNA expression (Figure 7e).

### 3.5. Phyto-Sesquiterpene Lactones Inhibits the Growth of TNBC Cells In Vivo

**Figure 8:**
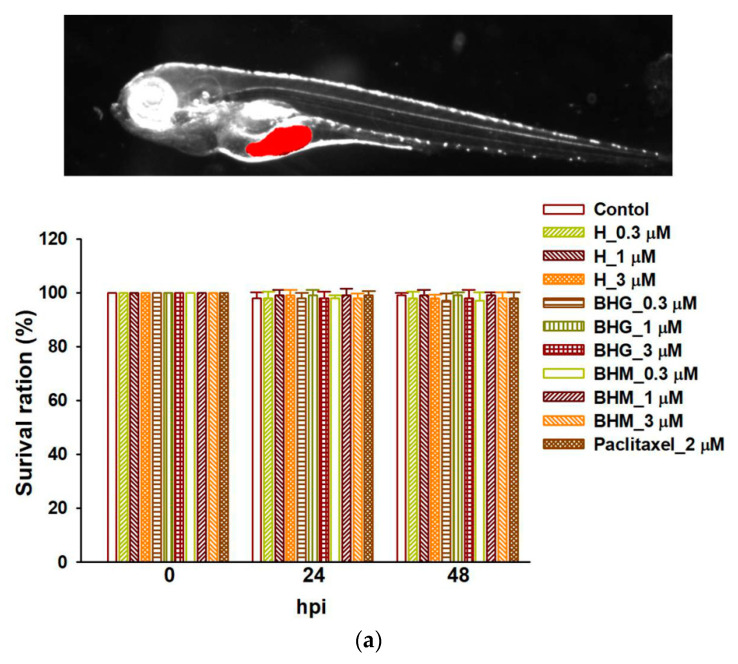

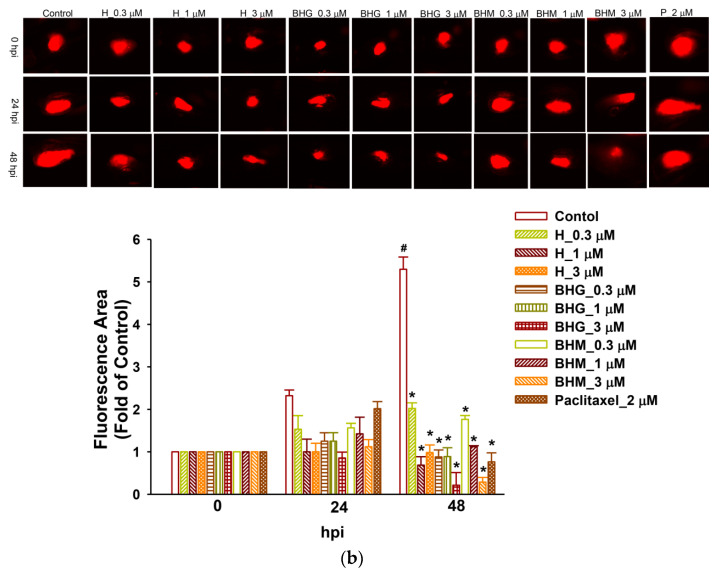
Helenalin, BHM, and BHG suppressed TNBC tumor growth in the xenotransplantation model. (**a**) The intensity of fluorescence was proportional to tumor size. There was no observable toxicity at 48 hpi after each compound treatment. P denoted as paclitaxel (positive control). (**b**) The MDA-MB-231 xenotransplantation model was treated in the presence and absence of test compounds, and helenalin, BHM, and BHG significantly inhibited tumor growth after 48 h of treatment. # *p* < 0.05 compared with the control; * *p* < 0.05 compared with control cells treated with test compounds. hpi: hours post-treatment or post-injection.

A zebrafish xenograft model with Dil-marked MDA-MB-231 cells was performed to confirm the tumor suppression effect of helenalin, BHM, and BHG on TNBC in vivo. The fertilization embryos of zebrafish were randomly divided into 0.3, 1, or 3 μM of helenalin, BHM, and BHG, 2 μM of paclitaxel (positive control), and untreated control group treatments. No toxicity was observed with the indicated concentrations treatment (Figure 8a). Nevertheless, test compounds exerted stronger potency on tumor suppression than the control after 48 h of treatment (Figure 8b). Helenalin, BHM, and BHG were potentially effective and safe compounds for TNBC treatment in vivo.

## 4. Discussion

Until now, chemotherapy was the main treatment option for advanced TNBC. Recently, the U.S. Food and Drug Administration (FDA) approved atezolizumab, an immune checkpoint inhibitor, for use in combination with paclitaxel for advanced-stage patients [34]. Unfortunately, the frequent relapse of tumors and intolerance to the systemic side effects of chemotherapy remain major obstacles for the prognosis of TNBC. Therefore, innovative therapeutic strategies that directly affect cancer cells are increasing in need. MYC dysregulation plays a crucial role in the tumorigenesis of various cancers, including TNBC. In clinical studies, TNBC patients with MYC overexpression show poor outcomes [35]. However, novel compounds that selectively target MYC have not been clarified and explored in TNBC. In the present study, we aimed to identify molecules that suppress TNBC cell proliferation by affecting the MYC pathway and are sourced from natural products. Our study demonstrated that MYC expression was relatively high in TNBC patients compared to other types of breast cancer in the METABRIC. We also observed that the natural sesquiterpene lactone helenalin and its derivatives BHM and BHG exhibited more potent and promising inhibiting ability toward MDA-MB-231 cells, and similar results were observed in zebrafish xenograft models. Mechanistic studies have shown that helenalin exerts inhibition mainly by inducing cell cycle arrest in the G2/M-phase. It is likely that the inhibition of MYC also blocks its downstream proteins CDC25 and CDK1 because CDK1 must enter the nucleus during the G2/M transition process and be activated by CDC25 phosphatase. BHM and BHG markedly induced cell apoptosis, as demonstrated by the increased levels of ROS, caspase 9 activity, and subG1-phase DNA. Most prior studies on the relationship between MYC and TNBC focused on cancer stem cell properties, finding that MYC activated the Wnt/β-catenin pathway and then initiated cancer stem cell amplification and tumorigenesis [36]. A previous study also demonstrated the presence of a synthetic-lethal interaction between MYC overexpression and CDK1 inhibition [10]. In the current study, we exploited the property of MYC overexpression in TNBC to identify three phytocompounds that repressed MYC/CDC25/CDK1 signaling. Moreover, these three compounds activated *p53*, followed by downregulation of MYC protein level, resulting in cell growth arrest and apoptosis. Helenalin, BHM, and BHG inhibit tumor growth and may prevent multidrug resistance in TNBC by targeting key signaling molecules and ABC transporters, with cytotoxicity varying by target expression levels.

Helenalin and BHM have been developed as antileukemic agents that inhibit protein and nucleic acid synthesis, while BHG is mainly associated with the inhibition of DNA topoisomerase II [27,37,38]. Structurally, both BHM and BHG are dimers of helenalin, in which two helenalin molecules are connected via a defined chemical linker. Biological assays demonstrated that BHM and BHG exhibit enhanced cytotoxicity compared to helenalin alone in specific cancer cell lines, notably in the multidrug-resistant MDA/doc cell line. These results suggest that helenalin dimerization may enhance biological activity by improving molecular stability, facilitating cellular uptake, or promoting multivalent interactions with molecular targets. This structure–activity relationship (SAR) insight not only supports the observed outcomes but also provides a foundation for the rational design of next-generation helenalin-based anticancer agents. Limited studies have been done to investigate the effect of helenalin and its derivatives on TNBC. Recently, helenalin demonstrated antiproliferative activity in another TNBC cell line, HBL100, but the article did not provide more detail about the cytotoxic mechanism [39]. Some studies have mentioned the anticancer activity of sesquiterpene lactones, and other sesquiterpene lactone analogues have been identified as effective anti-TNBC agents [23]. Alantolactone, the main component of the roots of *Inula helenium*, exhibits various biological properties, including anti-inflammation and apoptotic induction. Alantolactone significantly suppressed translocation to the nucleus and DNA binding of STAT3 and *NF-κB* in MDA-MB-231 cells [22]. Another study illustrated that a natural sesquiterpene lactone, eupalinolide, also acted as a STAT3 inhibitor against MDA-MB-468 cell survival [25]. Similarly, our test compounds exhibited STAT3 suppression and further inhibited the expression of its downstream target protein, MYC, in MDA-MB-231 cells. These data suggested that STAT3 may be used as a promising therapeutic target for TNBC patients along with MYC.

Functional assays showed that helenalin, BHM, and BHG inhibited the efflux activity of *ABCB1* and *ABCG2* transporters in the respective overexpression cells. ABC transporter overexpression is a leading cause of cancer treatment failure. Chemotherapeutic agents, such as paclitaxel, docetaxel, and doxorubicin, are current standard treatments for TNBC and substrates of *ABCB1* and *ABCG2* transporters.

Our test compounds have both selective cytotoxicity to TNBC cells and inhibition of the efflux of chemotherapeutic drugs. Notably, treatment with a combination of helenalin, BHM, or BHG with doxorubicin or docetaxel revealed a strongly synergistic effect, resulting in better anti-proliferative effects than drug treatment alone. Previous studies have not addressed whether BHM and BHG exhibit superior efficacy compared to helenalin in cell lines overexpressing *ABCB1* or *ABCG2*. In contrast, our findings demonstrate that the dimerization of helenalin enhances synergistic cytotoxicity in *ABCB1*- and *ABCG2*-overexpressing cell lines, as well as in the multidrug-resistant MDA/doc cell line. This improved efficacy may be attributed to the increased number of alkylating centers within the dimers, which potentially facilitate stronger interactions with cellular targets. Additionally, the dimers may undergo intracellular breakdown, releasing active monomeric units to enhance the therapeutic effect further. These results suggest that helenalin, BHM, and BHG hold promise as either monotherapy or in combination with standard chemotherapeutic agents for the treatment of TNBC and may contribute to the prevention of MDR development. Even so, the present study still has some limitations that cannot be overlooked. For example, enrolling different TNBC cell lines and using stable MYC-overexpression clones to verify selective cytotoxicity. In addition, although the MYC inducing G2/M arrest by regulating CDK1 and p21 have been respectively demonstrated in previous studies, indirect mechanisms are needed for further investigation since MYC involves many molecular cell signaling.

## 5. Conclusions

Together, this study demonstrated for the first time that helenalin, BHM, and BHG can inhibit tumor cell growth in vitro and regulate various signaling molecules, including STAT3, MYC, CDC25, CDK1, p53, and p21, and can potentially prevent the development of multidrug resistance in TNBC via ABC transporter inhibition. Considering the clinically collected evidence, MYC could be exploited as a potential therapeutic target for TNBC, and helenalin, BHM, and BHG may provide leading structures to be developed as novel agents in future TNBC treatment.

## Figures and Tables

**Table 1:** Cytotoxicity of helenalin, BHM, and BHG on different cancer cell lines.

Compound	MDA-MB-231	MDA-MB-453	MDA-MB-361	MCF7	HepG2	NCI-H460	MDA/doc
	IC_50_ (μM)						
Helenalin	0.63 ± 0.03	5.71 ± 0.67 *	7.63 ± 0.20 *	1.75 ± 0.12 *	2.80 ± 0.07 *	4.52 ± 0.07 *	7.69 ± 0.19 *
BHM	0.07 ± 0.001	3.24 ± 0.27 *	5.34 ± 3.34	0.58 ± 0.03 *	0.69 ± 0.002 *	0.99 ± 0.16 *	0.73 ± 0.03 *
BHG	0.55 ± 0.003	4.86 ± 0.73 *	16.33 ± 0.39 *	2.50 ± 0.12 *	3.60 ± 0.004 *	4.29 ± 0.09 *	0.86 ± 0.06 *
Paclitaxel	0.06 ± 0.01	N/A	N/A	N/A	N/A	N/A	90.56 ± 3.92 *

Statistical differences were evaluated by the ANOVA followed by post hoc analysis (Tukey’s test). * denoted *p* < 0.05 as compared to IC_50_ of MDA-MB-231. N/A: Not applicable. Data presented as mean ± standard error of at least three experiments, each in triplicate.

## Data Availability

The data that support the findings of this study are available from the corresponding author upon reasonable request.

## Supplementary Materials

The following supporting information can be downloaded at: https://www.mdpi.com/article/10.3390/cancers17081321/s1, Full pictures of the Western blots in Figure 3b.

## Abbreviations

The following abbreviations are used in this manuscript:

ABC	ATP-binding cassette
BCRP	Breast cancer resistance protein
BHG	Bis(helenalinyl)glutarate
BHM	Bis(helenalinyl)malonate
CDC25	Cell division cycle protein 25
CDK1	Cyclin-dependent kinase 1
ELISA	Enzyme-linked immunosorbent assay
ER	Estrogen receptor
FTC	Fumitremorgin C
HER2	Human epidermal growth factor receptor type 2
MDR	Multidrug resistance
MRP1	Multidrug resistance protein 1
PARP	Poly (ADP-ribose) polymerase
PBS	Phosphate-buffered saline
P-gp	P-glycoprotein
PI	Propidium iodide
PR	Progesterone receptor
ROS	Reactive oxygen species
SRB	Sulforhodamine B
STAT3	Signal transducer and activator of transcription 3
TCA	Trichloroacetic acid
TNBC	Triple-negative breast cancer
