# Supplementary figures and images for: Phyto-Sesquiterpene Lactones Prevent the Development of Multidrug Resistance in TNBC via ABC Transporters Inhibition and STAT3/MYC Signaling
Source: Cancers (Basel). 2025 Apr 14;17(8):1321. doi: 10.3390/cancers17081321 (PMC12026016; doi:10.3390/cancers17081321)

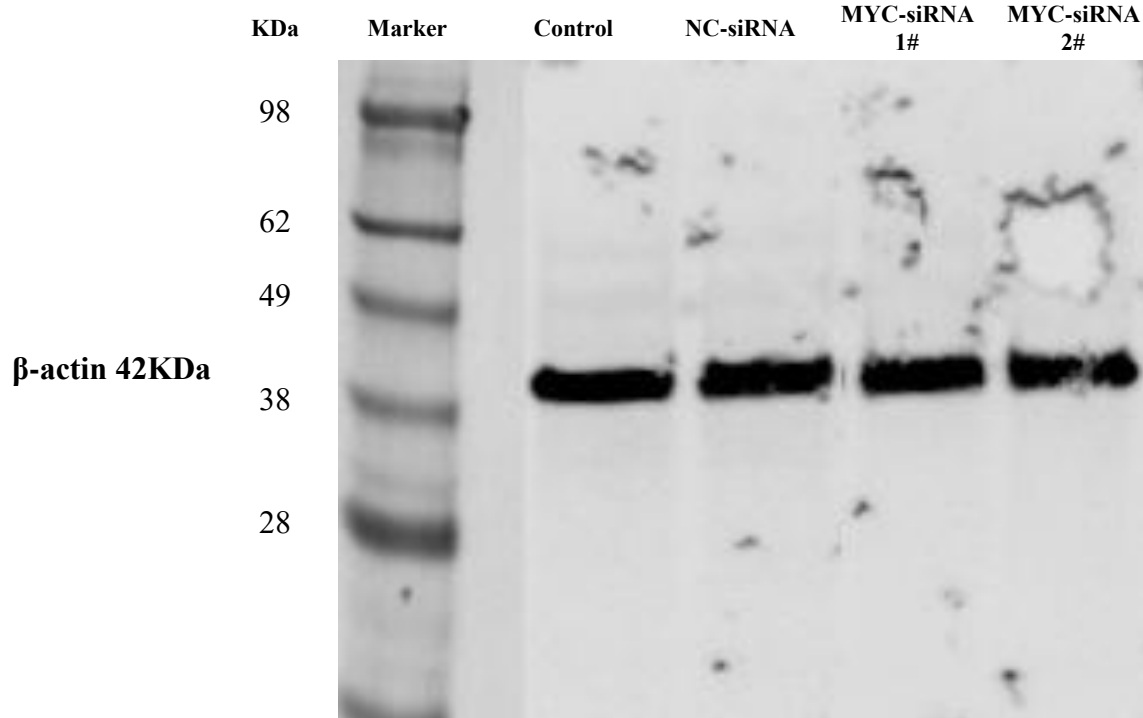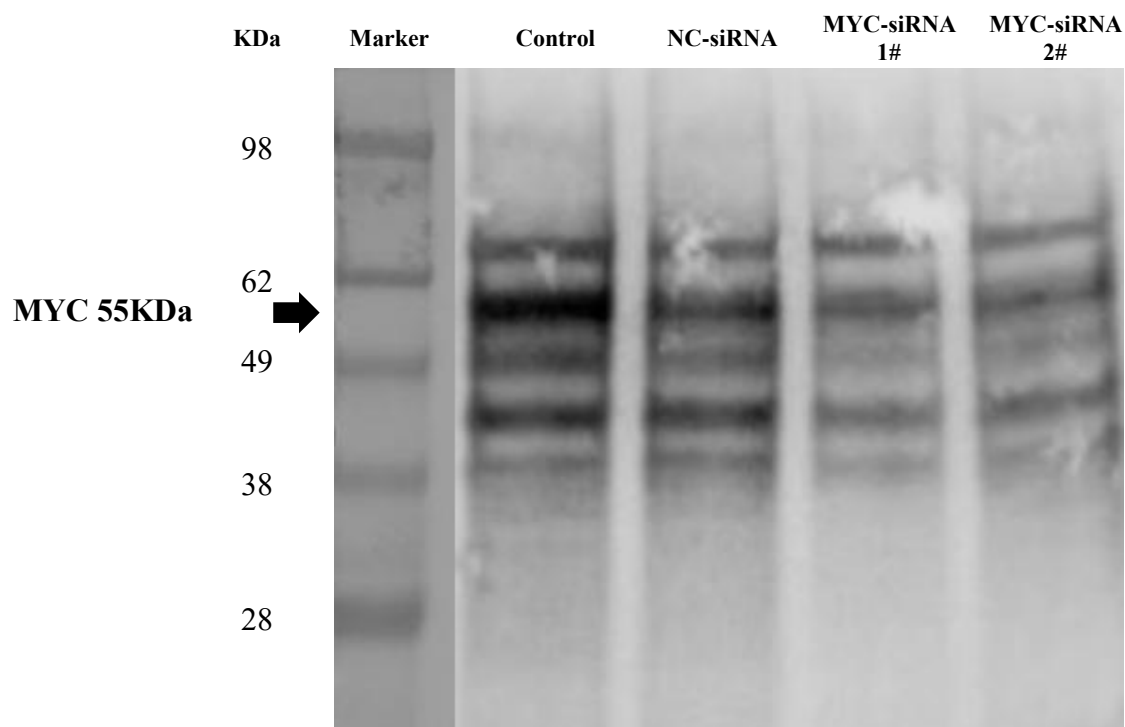

Original western blot for Figure 3b

Supplement: Supplementary file 1 [file cancers-17-01321-s001.zip › cancers-3537347-supplementary.pdf]
